# Prognostic factors for successful extubation in newborns with congenital diaphragmatic hernia

**DOI:** 10.3389/fped.2025.1530467

**Published:** 2025-01-27

**Authors:** A. Rannebro, C. Mesas-Burgos, U. Fläring, S. Eksborg, J. Berner

**Affiliations:** ^1^ Department of Pediatric Perioperative Medicine and Intensive Care, Karolinska University Hospital, Stockholm, Sweden; ^2^ Department of Pediatric Surgery, Karolinska University Hospital, Stockholm, Sweden; ^3^ Department of Women’s and Children’s Health, Karolinska Institutet, Stockholm, Sweden; ^4^ Department of Physiology and Pharmacology, Karolinska Institutet, Stockholm, Sweden

**Keywords:** congenital diaphragmatic hernia, neonatal, weaning, extubation failure, paediatric intensive care

## Abstract

**Introduction:**

Neonates with congenital diaphragmatic hernia (CDH) have an associated high mortality and morbidity. The European CDH EURO consortium has developed guidelines for initial and perioperative ventilatory management. There are, however, no recommendations on how to wean these patients from the ventilator. Extubation failure is more frequent in this group of patients than in other neonates. The aim of this study was to describe patient characteristics and risk factors for failed extubation and to evaluate predictive factors for successful weaning.

**Methods:**

We performed a retrospective study in a single centre tertiary pediatric intensive care unit in Stockholm, Sweden. CDH-patients (*n* = 38), aged 0–28 days, with extubation events were identified from 2017 to 2019. Eight patients (21.1%) needed reintubation within 24 h after the first extubation attempt. Patient demographics, surgical repair with patch, oxygenation saturation index (OSI), rapid shallow breathing index (RSBI), ventilatory settings, fluid balance and sedation on the day of extubation were recorded.

**Results:**

Patients in the failed extubation group (FE) had lower birth weight (*p* < 0.05), surgical patch repair (*p* < 0.05), longer length of stay in intensive care (*p* < 0.05), longer time on the ventilator (*p* < 0.05) and other comorbidities (*p* < 0.001). Using logistic regression we identified OSI, RSBI and inspiratory pressure (Pinsp) as factors predicting a successful extubation, AUCROC 0.95 (95% CI: 0.87 to 1.00). Patients in the FE-group had significantly more often pulmonary hypertension requiring treatment (*p* < 0.05), a higher fraction of inspired oxygen (FiO_2_) (*p* < 0.05) and hypercapnia (*p* < 0.001) prior to extubation and an oxygen demand exceeding 40% two hours after extubation (*p* < 0.05).

**Conclusion:**

Useful predictors of successful extubation in CDH patients are OSI, RSBI and Pinsp. Low birth weight, patch repair and comorbidity also appear to be important factors. Prospective studies are required to confirm findings in the present study.

## Introduction

Congenital diaphragmatic hernia (CDH) is a rare condition with an incidence of approximately 40 cases per 100,000 births (1). Despite advancements in pediatric critical care, it remains a significant challenge for clinicians, with an overall mortality of 28% over the last 25 years (2). The majority of CDH patients require invasive ventilation immediately after birth to maintain adequate gas exchange (3, 4). This is due to pulmonary hypoplasia, which occurs to varying degrees because of abdominal contents herniating into the thorax during fetal life. The extent of lung hypoplasia depends on both the size of the herniation and the gestational timing of the herniation (5).

Corrective surgery is usually performed after a few days of stabilization (1, 3). Postoperatively, mechanical ventilation is required in almost all patients for a variable length of time. However, it is critical to minimize the duration of invasive ventilation due to the associated risks for ventilator-induced lung injury (6), ventilator-associated pneumonia (7, 8), higher morbidity and mortality and also the increased length of stay (LOS) and hospital costs (9–11).

Weaning refers to the gradual transition from full invasive ventilatory support to spontaneous breathing, with or without non-invasive ventilatory support, such as continuous positive airway pressure (CPAP) or high flow nasal cannula (HFNC). Optimizing the timing of weaning and extubation is important to reduce the risk of complications from invasive ventilation (12) while avoiding the risks of reintubation (13, 14).

Failed extubation (FE) has been well-studied in the pediatric intensive care population, with reported incidences ranging between 5%–10% (9, 15). However, there is limited literature on the incidence of FE specifically in newborns with CDH. A retrospective study of CDH patients by Schroeder et al. reported an incidence of 35% (16). In general, existing weaning protocols are few and specific guidelines for weaning CDH-patients are lacking.

The intensive care of CDH patients after corrective surgery remains highly challenging. It is of outmost importance to avoid desaturation episodes, pulmonary hypertension crisis, trauma to the upper airways, and ventilator induced lung- injury, among other complication.

In Sweden, the care of CDH patients has been centralized since 2017, as mandated by the Swedish National Board of Health and Welfare, and the aim of this study was to investigate the incidence of FE at our high volume CDH centre, and to identify variables that could predict a successful extubation in this particular group of patients.

## Materials and methods

This is a retrospective, single centre study. Data was collected from all newborn patients with CDH admitted to our PICU at the Astrid Lindgren Childreńs Hospital, Karolinska University Hospital between January 1, 2017 and December 31, 2019. Patients were identified using the Swedish patient administrative system for intensive care units (PAS-IVA, Otimo Data AB, Kalmar, Sweden) and the Swedish Intensive Care Registry (SIR).

We reviewed patient records and data from our patient data management system (Centricity Critical Care, GE Health Care). Inclusion criteria were the presence of CDH, age 0–28 days at admission to PICU and the need for invasive ventilatory support. Exclusion criteria were death during the period of ventilatory support in the PICU, extubation as a part of withdrawal of treatment/end of life and respiratory failure due to neurological disease.

Patients were divided into two groups: (1). Successful extubation (SE; control group) including patients with a successful first extubation attempt defined as no reintubation within 24 h. Second group; Failed extubation (FE; study group) including patients requiring reintubation within 24 h of the first extubation attempt.

Demographic data included variables such as birth weight, sex, gestational age, congenital cardiac malformation, and persistent pulmonary hypertension of the newborn (PPHN) were assessed. Comorbidities other than CDH (neurological, pulmonary, renal, gastrointestinal, ongoing pneumonia or other infections) were also recorded. CDH characteristics were collected [side of the defect, observed/expected lung-head ratio (O/E LHR%), patch or primary repair]. Date and time of start and end of invasive ventilatory support, findings on chest x-ray (performed 0–48 h before extubation), including presence of infiltrates or atelectasis were also collected.

Sedation and analgesia score (Comfort B), sedative and analgesic doses, circulatory and respiratory variables, presence of vasoactive and/or pulmonary hypertension treatment and ventilatory settings were recorded 1 h prior to morning blood gas taken on the day of extubation. PaO_2_/FiO_2_%-index (PFI), oxygenation index (OI, calculated as [(FiO_2_xmean airway pressure)/PaO_2_ (converted to mmHg)]x100), oxygenation saturation index (OSI, calculated as FiO_2_x100/SpO_2_), rapid shallow breathing index [RSBI, calculated as breaths per minute/(tidal volume (ml)/ bodyweight (kg))] were calculated from the obtained data. These data were tested in monovariate analysis for successful/failed extubation and the three variables with best prediction were combined in logistic regression analysis.

Presence and type of non-invasive ventilatory support after extubation was recorded as CPAP, HFNC or other type of support i.e., flow-by oxygen through a mask, low flow nasal cannula or none and also the need of FiO_2_ > 0.4 2 h post extubation. Reintubation within 24 h was noted.

## Statistics

Mann–Whitney *U*-test was used for comparison of two unrelated populations. Models' discrimination was evaluated using c-statistics (ROC analyses).

Sensitivity and specificity of the ROC curve were evaluated by the Youden index (17). Calibration belt analyses were performed by RStudio 2022.02.3 Build 492 (18).

Statistical analysis was carried out using MS Excel (Microsoft Corporation, Redmond, WA, USA) and GraphPad Prism version 5.04 and version 10.2.0 (GraphPad Software inc. San Diego, CA, USA).

All reported *p*-values are from two-sided tests. A *p*-value less than 0.05 is considered significant.

### Ethics

The study was approved by the Swedish Ethical Review Authority (Dnr 2021-05551-01).

## Results

**Figure 1:**
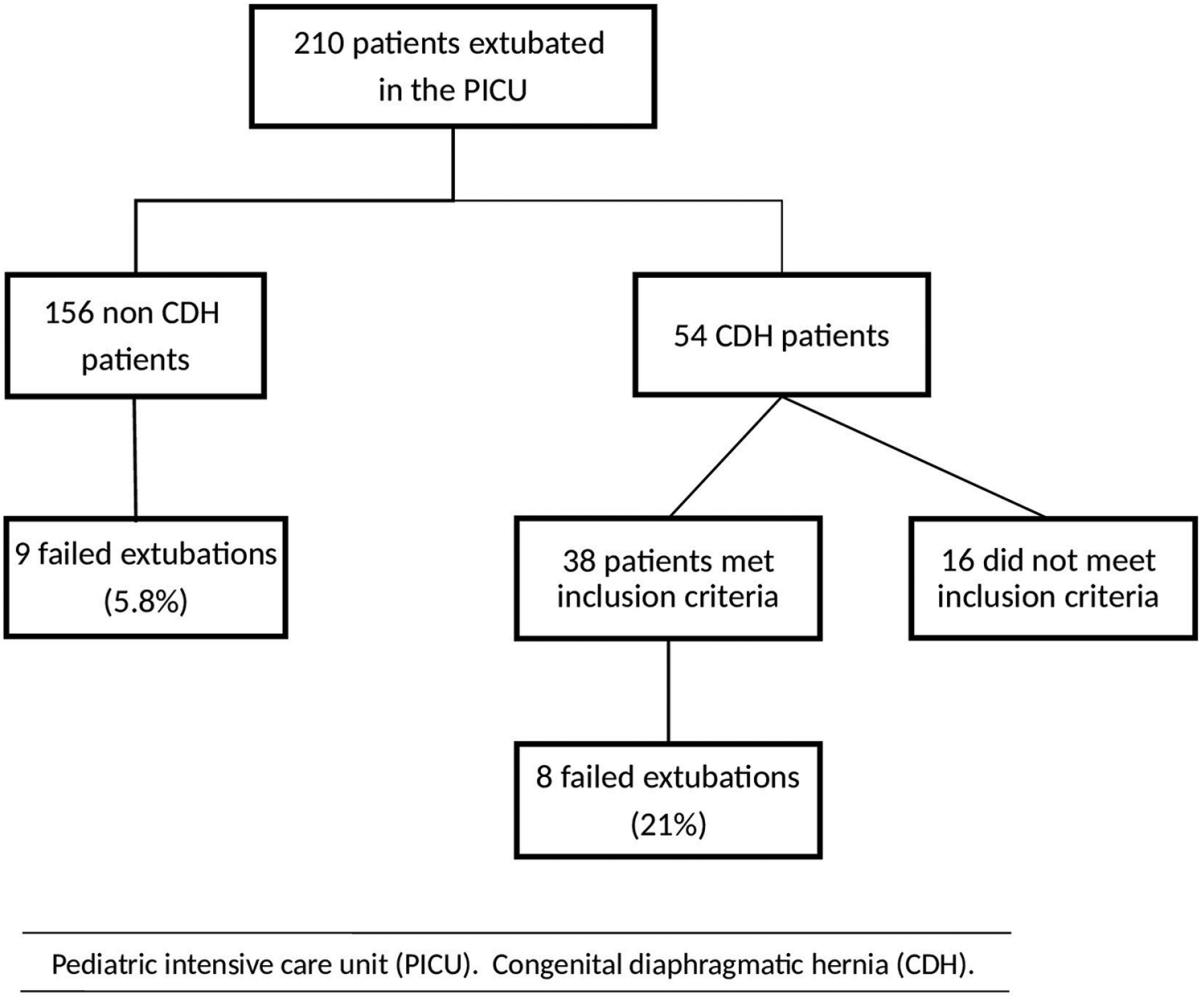
Flowchart showing patients on invasive ventilation in the PICU during years 2017–2019.

CDH patients (*n* = 54) were evaluated during the study period; 38 patients fulfilled the inclusion criteria and 16 did not meet the inclusion criteria (eight patients had never been to the PICU, four patients were older than one month on admission to the PICU, one patient was born in 2016, two patients did not have an extubation attempt in the PICU (foreign patients transferred back to referring hospital still on the ventilator), one patient was never on the ventilator during PICU care (Figure 1).

Eight patients (21.1%) required reintubation within 24 h of the first extubation attempt, and two of these eight patients subsequently failed a second extubation attempt. No patient failed more than twice.

Demographic data at the time of the first extubation attempt are presented in Table 1. Patients who failed extubation had significantly lower birth weight, surgical patch repair, longer LOS, longer time on mechanical ventilation and more associated comorbidities.

**Table 1:** Demographic data at first extubation attempt.

Variables	All patients	Group SE	Group FE	*p* value	Missing data
Number of patients	*n* = 38	*n* = 30 (78.9%)	*n* = 8 (21.1%)		
Weight (birth weight or weight at admission, kg)	3.2 (2.8–3.4)	3.3 (2.9–3.5)	2.8 (2.4–3.1)	0.0302	
Males	25 (65.8)	24 (92.3)	1 (12.5)	0.0009	
Gestational age (week)	38 + 0	38 + 3	37 + 3	0.2244	1 (SE)
CDH left sided	27 (71)	22 (73.3)	5 (62.5)	0.7661	
O/E LHR (%)	47.5 (36.5–52.9)	50.0 (37.5–55.2)	38.0 (28.5–50.0)	0.2125	20 (17 SE; 3FE)
Patch repair	26 (68.4)	18 (60)	8 (100)	0.0385	
VOC comorbidity	1 (5.3)	0 (0)	1 (12.5)	0.3812	
Other comorbidity	7 (18.4)	3 (10)	4 (50)	0.0245	
PIM3 (%)	9.9 (6.9–15)	9.9 (7.0–15)	9.8 (6.0–18)	0.9999	
PICU stay (days)	18.1 (7.3–26.8)	11.6 (7.2–24.7)	26.1 (24.2–37.4)	0.0414	
Fluid balance (%)	2.3 (−1–7.1)	1.7 (−2–6.9)	5.4 (0.5–9.6)	0.1714	
Total invasive ventilation (h)	147 (37.0–291)	141 (37.0–204)	340 (187–481)	0.0208	
ECMO treatment *n* (%)	6 (15.8)	3 (10)	3 (37.5)	0.0940	

SE, successful extubations; FE, failed extubations. Missing data is presented as individuals per group. Congenital diaphragmatic hernia (CDH), observed estimated lung area to head circumference ratio (O/E LHR), the ratio is calculated by dividing the observed lung area by the estimated lung area, and it is expressed as a percentage calculated as (Estimated Lung Area for gestational age/Observed Lung Area) × 100. Pediatric index of mortality (PIM) 3 is a scoring system used to predict the risk of mortality in critically ill children. Pediatric intensive care unit (PICU). Fluid balance is calculated from weight at admission and actual weight. Total invasive ventilation time during PICU stay. Extra corporal membrane oxygenation (ECMO). Data are presented as median (interquartile range) or absolute number (n) with percentage.

Treatment and scores at the first extubation attempt are presented in Table 2. There were significantly more females in the FE group compared to the SE group (87.5% vs. 7.7%, respectively), and other comorbidities except heart malformations were more common in the FE group (50% vs. 10%, respectively). No differences were found in gestational age, side of the defect, PIM3, ECMO-treatment or cardiac malformations. Analgosedation was similar between the FE and SE groups: intravenous opioid treatment (8.3 vs. 2.4 µg/kg/h, respectively), and midazolam (21.6 vs. 1.5 µg/kg/h, respectively), nor was there any difference in the comfort B score. After extubation, 57.8% received CPAP (87.5%) in FE group and 50% in SE group, *p* = 0.1056) and 36.8% received HFNC (0% in FE- group and 46.7% in SE-group, *p* = 0.0335). Oxygen requirement more than 40% two hours after extubation was significantly higher in the FE group as compared to the SE group (62.5% and 16.7%, respectively, *p* = 0.0186).

**Table 2:** Treatment and scores at first extubation attempt.

Variables	All patients	Group SE	Group FE	*p* value
Number or patients *n*	38	30	8	
Comfort B	12.0 (11.0–13.0)	12.0 (11.0–13.2)	12.0 (10.5–12.0)	0.6602
PPHN treated patients *n* (%)	16 (42.1)	10 (33.3)	6 (75.0)	0.0498
FiO_2_%	26.5 (21.0–30.0)	25.0 (21.0–28.5)	30.0 (28.5–35.0)	0.0163
RSBI	9.6 (7.4–12)	9.2 (7.0–12)	12 (11–17)	0.0014
Sedative drugs, 24 h prior extubation day				
Morphine/Ketobemidon (µg/kg/h)	4.2 (0.3–7.7)	2.4 (0.3–7.1)	8.3 (1.8–9.6)	0.1253
Midazolam (µg/kg/h)	3.2 (0–43.6)	1.5 (0–43.6)	21.6 (0–45.6)	0.5725
alfa-2 agonist *n* (%)	38 (100)	30 (100)	8 (100)	N/A
Ventilatory support after extubation				
CPAP *n* (%)	22 (57.8)	15 (50)	7 (87.5)	0.1056
HFNC *n* (%)	14 (36.8)	14 (46.7)	0 (0)	0.0335
Other *n* (%)	2 (5.3)	1 (2.6)	1 (12.5)	N/A
O_2_ demand >40% after extubation *n* (%)	13 (26.3)	5 (16.7)	5 (62.5)	0.0186

SE, successful extubations; FE, failed extubations. Comfort B—pain and sedation assessment tool for children; Score of 6–10: Deep sedation or no distress; 11–22: adequate sedation or mild distress; 23–30: moderate to severe distress. Persistent pulmonary hypertension of the newborn (PPHN) treatment (Nitric oxide, sildenafil, milrinone alone or in combination). Fraction of inspired oxygen (FiO_2_). Rapid shallow breathing index (RSBI) calculated as breaths per minute/ [tidal volume (ml)/ bodyweight (kg)]. Sedative drugs shows the total amount given the last 24 h before extubation day. Continuous positive airway pressure (CPAP). High flow nasal cannula (HFNC). Other ventilatory support refers to oxygenation by nasal cannula or open mask. Oxygen demand of more than 40% at the gas mixer. Data are presented as median (interquartile range) or absolute number (n) with percentage SE.

**Figure 2:**
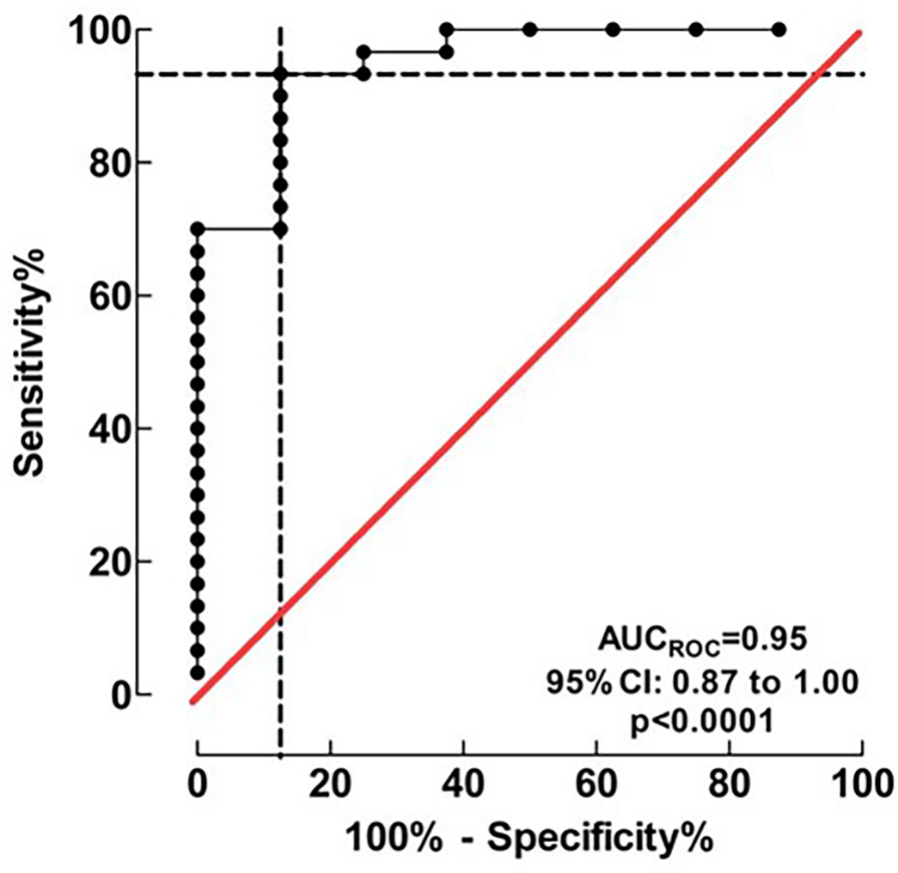
Discrimination of the logistic regression model for successful extubation. Three factors (OSI, RSBI and Pinsp), combined in the logistic regression model, proved a high discriminating ability to identify patients in the FE and SE groups. AUCROC value was 0.95 (95% CI: 0.87 to 1.00) indicating an excellent accuracy of discrimination.

**Figure 3:**
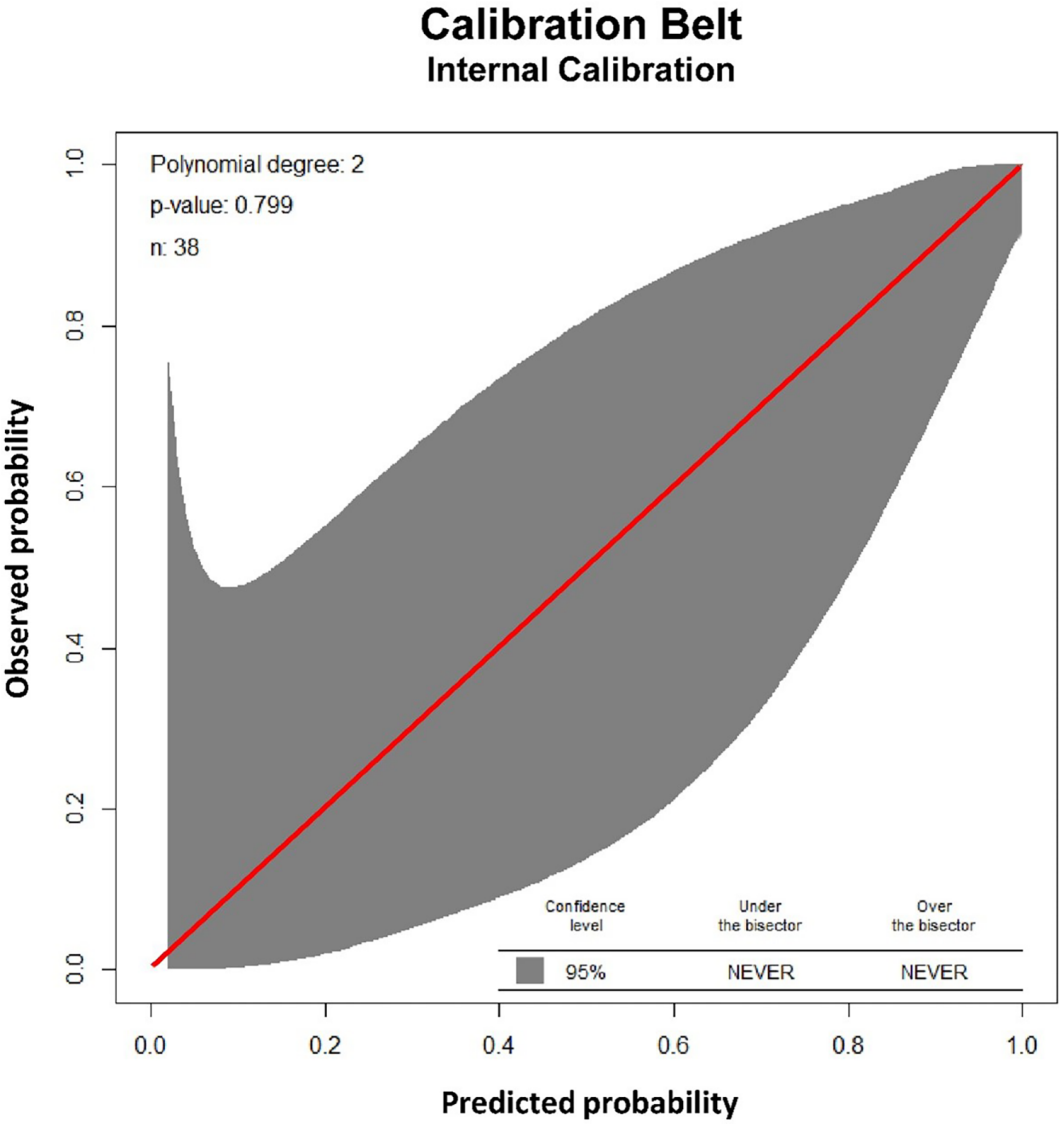
Validation of the logistic regression model for successful extubation. The calibration belt analysis of the logistic model including three factors (oxygenation saturation index, rapid shallow breathing and peak inspiratory pressure) using internal calibration showed a *p*-value of 0.799, i.e., the slope of the observed risk and predicted probability did not deviate from unity. All data were within the 95% confidence interval (shadow area).

Physiological parameters at first extubation attempt are summarized in Table 3. Three factors with the lowest *p*-values in separate monovariate analysis for successful extubation, (OSI, RSBI and Pinsp), combined in logistic regression, proved to have a high discriminating ability to identify patients in the FE and SE groups. The area of the ROC curve (AUCROC) was 0.95 (95% CI: 0.87 to 1.00; *p* < 0.001), Figure 2. The calibration belt analysis with internal calibration showed a *p*-value of 0.799, i.e., the slope of the observed risk and predicted probability did not deviate from unity. All data were within the 95% confidence interval, Figure 3.

**Table 3:** Physiological parameters at first extubation attempt.

Variables	All patients	Group SE	Group FE	*p* value	Missing data
Number of patients	38	30	8		
OI	15.4 (13.7–20.5)	16.1 (13.5–20.0)	15.4 (13.6–23.7)	0.9999	9 (1FE; 8 SE)
OSI	2.1 (1.8–2.7)	2.0 (1.7–2.3)	3.2 (2.4–4.0)	0.0006	
PFI	34.4 (31.6–40.6)	35.6 (32.6–41.2)	30.9 (24.1–40.0)	0.1076	9 (1 FE; 8 SE)
MAP (cmH_2_O)	7.0 (6.7–8.6)	7.0 (6.7–8.4)	7.9 (6.4–9.5)	0.5432	
Pinsp (cm H_2_O)	17.4 (14.8–19.0)	16.1 (14.5–18.1)	20.1 (18.5–22.1)	0.0003	
SpO_2_ preductal (%)	97.4 (96.6–98.6)	98.0 (96.7–98.6)	97.0 (95.4–98.6)	0.1646	
PaO_2_ (kPa)	9.7 (8.1–10.2)	9.8 (8.3–10.2)	8.4 (8.1–10.8)	0.6994	9 (1FE; 8 SE)
PaCO_2_ kPa	6.3 (5.7–7.2)	6.2 (5.4–6.9)	7.4 (7.2–7.8)	0.0006	8 (1 FE; 7 SE)

SE, successful extubations; FE, failed extubations. Missing data is presented as individuals per group. Oxygenation index (OI) calculated as [(FiO_2_xmean airway pressure)/PaO_2_ (converted to mmHg)]x100. PaO_2_ (mmHg)/FiO_2_ index (PFI), mean airway pressure (MAP). These two variables have a high proportion of missing data in both groups, reflecting poor access to arterial lines. Oxygenation saturation index (OSI) calculated as FiO_2_x100/SpO_2_. Inspiratory pressure (Pinsp). Saturation of peripheral oxygen (SpO_2_), partial pressure of oxygen in arterial blood (PaO_2_), partial pressure of carbon dioxide in arterial blood (PaCO_2_). These two variables also have a high proportion of missing data in both groups, reflecting poor access to arterial lines. Data are presented as median (interquartile range) or absolute numbers (n) with percentage.

## Discussion

Our study shows that it is possible to discriminate between successful and failed extubation within the first 24 h in CDH patients. Using logistic regression we identified OSI, RSBI and Pinsp as three predictive factors for successful extubation. Using these three factors in combination might be a useful tool for achieving successful extubation. The three factors reflect oxygenation, tidal volume and inspiratory pressure required to achieve effective ventilation. We used OSI because there were no missing data compared to 24% missing data for OI (not surprisingly 8 out of 9 were in the SE group, reflecting that not all patients still had an arterial line at the time for extubation). Furthermore, OSI is strongly correlated with OI (19, 20). Using a calibration plot belt we could see good agreement between our predictive model and FE (*p* = 0.799, Figure 2), which served as a model validation.

Mechanical ventilation is a cornerstone in neonatal and pediatric critical care. However, it is generally thought to be important to wean patients from the ventilator as early as possible to avoid associated complications. The timing of weaning is largely dependent on clinician judgement as several extubation readiness protocols have been shown to be inadequate (21, 22). Our interest was in this particular group of patients with CDH and mechanical ventilation with a suspected high incidence of FE. To our knowledge, guidelines for weaning CDH patients from mechanical ventilation are scarce or non-existent.

All infants in the FE group had a patch repair as compared to 60% in the SE group, significantly longer PICU stay (11.6 vs. 26.1 days) and also significantly longer total invasive ventilation time (140.7 vs. 340.2 h). These findings are consistent with a previous study by Schroeder et al. and Brindle et al. (16, 23), showing higher morbidity in patients requiring patch repair (16). In addition, this study also reported that ECMO treatment and low estimated lung area to head circumference ratio (LHR) were indicators of FE, whereas in our cohort 37.5% needed ECMO treatment in FE group and 10% non ECMO in SE group (*p* = 0.0940) not reaching statistical significance. Regarding LHR variable we had missing data in 20 patients and therefore cannot draw any conclusions in comparison.

When analysing the type of ventilatory support after extubation we found that 87.5% were on CPAP and 62.5% had an O_2_ demand above 40% in the FE group, the latter may reflect which is likely due to be too early extubation attempts and may be an indication for the clinician to be cautious. Our recommendation is to assess readiness for extubation by evaluating OSI, RSBI and Pinsp. This ensures the patient is physiologically stable and ready for extubation. We recommend CPAP as the first line of respiratory support after extubation, to help maintain airway patency and support breathing in the immediate post-extubation period, particularly in patients with CDH. Step-wise change to HFNC after an evaluation of the patient´s respiratory status is clinical practice in transition after CPAP treatment in our unit.

Failed extubation in pediatric ICU patients has been reported in other studies to be around 5%–10% (9, 15). There is no standard definition for the observation time for failed extubation, ranging from 24, 48 to 72 h. Our clinical practice and Swedish national quality registry define FE as occurring within 24 h. Gaies et al. (24) reported in their large study on almost 1,500 pediatric cardiac patients that 71% of FE events occurred within 24 h and Edmunds et al. (15) reported that 70% of their cohort of 632 general PICU patients failed within 24 h, although the studies had different definitions, <48 and <72 h, respectively. We believe that our results are highly relevant for this cohort of patients, but this highlights the need for a consistent definition and design of prospective multi centre studies for more comparable results. It is also important to note that zero failed extubations are likely to prolong ventilator time and subsequent complications and is probably not something to aim for. Further research is needed to assess the appropriate number of failed extubations in CDH patients.

## Limitations

This study has limitations. It is a retrospective, high volume CDH specialized single-centre study with a small number of patients. The low sample size may affect the statistical analysis and findings must therefore be evaluated with caution even though we have strong significance. Our centre has been a national referral centre in Sweden since 2017 and is therefore experienced in the care of CDH patients. This may, of course, affect the generalizability of our findings for unexperienced low volume hospitals and can also affect the numbers of failed extubations. Some of the data are not measurements, but scores that are determined by the nursing staff, for example analgosedation scores such as Comfort B. Some parameters are not prioritized in the initial management of a newborn with CDH, for example birth weight, making it difficult to assess weight gain and fluid overload. Echocardiographic findings, such as signs of pulmonary hypertension and cardiac dysfunction were not recorded, only the presence or absence pulmonary hypertension medications and vasoactive agents. On the other hand the data collected is very robust due to the long experience of patient data management systems in our unit.

## Conclusion

A high incidence of FE in CDH patients (21%) was associated with OSI, RSBI and Pinsp among other factors. To our knowledge, this is the first study combining prognostic factors into a model to predict extubation failure in CDH-patients. These findings may contribute to the management of CDH-patients, and prospective studies are underway to confirm these findings.

## Data Availability

The original contributions presented in the study are included in the article/Supplementary Material, further inquiries can be directed to the corresponding author.
